# Design and Evaluation of a Novel Experimental Setup for Upper Limb Intermuscular Coordination Studies

**DOI:** 10.3389/fnbot.2019.00072

**Published:** 2019-09-06

**Authors:** Jeong-Ho Park, Joon-Ho Shin, Hangil Lee, Chan Beom Park, Jinsook Roh, Hyung-Soon Park

**Affiliations:** ^1^Department of Mechanical Engineering, Korea Advanced Institute of Science and Technology (KAIST), Daejeon, South Korea; ^2^Department of Neurorehabilitation, National Rehabilitation Center, Seoul, South Korea; ^3^Department of Biomedical Engineering, University of Houston, Houston, TX, United States

**Keywords:** intermuscular coordination, muscle synergy, upper limb, experimental set-up, mechanism design, performance evaluation

## Abstract

Motor disabilities limiting the mobility of limbs affect the quality of lives of people with neural injuries. Among various types of motor disabilities, abnormal intermuscular coordination is commonly observed from people with severe impairment. The concept of muscle synergy, defined as characteristic muscle co-activation patterns activated to produce complex motor behavior, has been applied to assess the alteration in intermuscular coordination in pathological populations. This study presents the development of a robotic system named KAIST upper limb synergy investigation system (KULSIS), for accurate measurement of intermuscular synergies while providing the convenient experimental setup. It provides full force/moment measurements for isometric force generation tasks at various upper limb postures and reaching tasks in a three-dimensional workspace. It is composed of: a three-degree-of-freedom gimbaled handle to adjust the orientation of the handle to accommodate potential hand-wrist deformity, a linear actuator that moves the handle for reaching tasks; a five-degree-of-freedom mechanism for positioning and adjusting the orientation of the linear actuator. The design was evaluated in terms of the workspace of the handle, mechanical stiffness and force/moment measurement accuracy. The position/force measurement is synchronized with electromyographic measurements. Muscle synergy patterns, activated during four isokinetic reaching motions, were also assessed as preliminary data using KULSIS from ten healthy subjects.

## Introduction

Neurological injuries result in limiting the activities of daily living and the quality of life. For example, hemiparetic stroke often manifests major motor issues such as spasticity, muscle weakness and stereotypical, abnormal motor coordination (Dewald et al., 2001; Macko et al., 2001). Among the motor impairments, abnormal motor coordination, namely “muscle synergy in stroke,” was described historically based on visual observation, rather than kinetic or electromyographic assessment (Twitchell, 1951; Brunnstrom, 1970). The later studies quantified the abnormal limb synergies in terms of the abnormal pair-wise muscle coactivation (Dewald et al., 1995) and torque coupling (Beer et al., 1999; Dewald and Beer, 2001). Relatively recent studies have quantified the impaired motor coordination in terms of multi-dimensional intermuscular coordination patterns, instead of pair-wise correlation of muscle activation, in the human upper extremities (UE) by applying dimensionality reduction techniques to the EMG signals collected from stroke survivors (Cheung et al., 2009, 2012; Roh et al., 2013, 2015; Tropea et al., 2013; García-Cossio et al., 2014; Li et al., 2017; Pan et al., 2018; Valk et al., 2019).

The previous studies of the human upper extremity focused on characterizing intermuscular coordination patterns of each individual task, for example, reaching (Cheung et al., 2009, 2012; Tropea et al., 2013; Li et al., 2017), isometric force generation (Roh et al., 2012, 2015), and movements of arm and hand (García-Cossio et al., 2014). Interestingly, the current literature has reported different mechanisms of alteration in intermuscular coordination depending on the type of motor tasks. Because of the difference in muscle selection (Steele et al., 2013) and inter-subject variability, it has been challenging to compare the mechanism of how stroke affects intermuscular coordination across diverse UE motor tasks under isometric conditions and in motions. These tendencies in the upper extremity study limit the understanding of the degree to which abnormal intermuscular coordination patterns are generalized across different motor tasks in the upper extremity post-stroke, which is essential to develop novel therapeutic strategies guided by altering the impaired coordination pattern widely used for different motor tasks to become similar to the normal pattern to improve motor function.

In order to examine the generalizability and specificity of intermuscular coordination patterns, a mechanical device can be used to implement both static and dynamic motor tasks of the human upper limb. However, a commercial device which is suitable for the assessment of motor coordination both under an isometric condition and in motion is relatively rare and often involves its own constraints to examine intermuscular coordination patterns in varying biomechanical conditions. For example, two different versions of KINARM (BKIN Technologies Ltd., ON, Canada; Scott, 1999), InMotion Arm (Bionik Laboratories, ON, Canada; Krebs et al., 1998), HapticMaster (Moogs FCS Inc., Netherlands; Van der Linde et al., 2002) and WAM (Barrett Technology Inc., MA, United States; Townsend and Guertin, 1999) are commercially available for assessment and/or therapy of motor disabilities after neural injuries. Among them, few planar devices, such as the two KINARM robots and InMotion Arm, allow the movement of a subject’s hand only in the horizontal plane. They cannot implement postures of different heights of the hand and reaching motions out of the horizontal plane. Other devices, such as the KINARM devices, HapticMaster and WAM can withstand only an external force below 100 N continuously or even instantly, which is much smaller than maximal force generation capacity of healthy adults. They usually adopt a multi-link arm structure to cover their workspace, but the structure is disadvantageous to endure the large force exerted at the end-point. Without additional mechanisms to fix the posture of the devices, large actuators with high torque capacities are required at their joints. In addition to the commercial robots, MACARM, a cable-driven haptic device had been developed (Mayhew et al., 2005) and adopted to several motor coordination studies under isometric conditions (Roh et al., 2012, 2013, 2015) and reaching (Beer R. F. et al., 2008). It features a large three-dimensional workspace implemented by the eight actuators located at the corners of its cubic frame. According to the technical report of MACARM (Beer R. et al., 2008), however, as the end-point of MACARM deviated from the center of the workspace, its position became unstable against the smaller force due to slack of the cable mechanism. In addition, it also has limitations of a large space requirement due to the frame structure and high complexity of the system involving many actuators.

The device, named KAIST upper limb synergy investigation system (KULSIS), provides a unique environment where the reaching path is matched between subject populations in comparison. It adopts a linear track along which subjects produce reaching movement. This constraint ensures a comparably similar end-point trajectory of reaching in both neurologically intact and pathological participants in comparison with minimal interference to the subject. While constraining the motion of the end-point, the pathological subject can still utilize impaired intermuscular coordination since alterations in joint kinematics of elbow and shoulder are allowed. Thus, potential alterations in intermuscular coordination patterns and their activation profile observed in a pathological group can be interpreted as the effects of the pathological condition during reaching movement in a well-controlled way.

This study proposes an experimental setup to examine coordination of upper limb muscles, which features a combination of single active DOF and five passive DOFs. The proposed structure of KULSIS supports a consistent examination of intermuscular coordination for both isometric force generation and constrained reach conditions in a large workspace of the human upper extremity. KULSIS can move a force/moment-measuring handle that a subject hold during measurement along a line in the workspace of upper limb to implement a variety of upper limb postures and the directional variances of reaching motion. During the subject performs given upper limb tasks implemented by KULSIS, force/moment at hand are measured as well as the movement of the hand. Activation of upper limb muscles and detailed kinematics of upper limb joints can be measured using commercial EMG and motion capture devices. Then, intermuscular coordination is analyzed by applying a non-negative matrix factorization (NNMF) algorithm (Lee and Seung, 2001) to a given EMG data. Effects of the altered intermuscular coordination on the subject’s motor performance can be investigated by performing a quantitative analysis of the motion and force measurements. This paper mainly presents the design of the KULSIS system and its evaluation in terms of (1) work volume of the handle, (2) deformation and mechanical stiffness against external loads, and (3) force/moment measurement accuracy. Section “Design of KULSIS” describes the design of KULSIS, and section “Evaluation of KULSIS” presents the method and the results of the evaluation. In addition to the evaluation of the device, intermuscular coordination measured by KULSIS from healthy participants is also presented. The results in section “Evaluation of KULSIS”, as well as the ways of possible design improvement, are discussed in section “Preliminary Intermuscular Coordination Assessment Using KULSIS”.

## Design of Kulsis

### Design Specifications of KULSIS

KAIST upper limb synergy investigation system constrains the trajectory of subjects’ hands during reaching tasks since how similar motions they perform is important for fair comparison of intermuscular coordination across subjects. Specifically, KULSIS allows the subject to move their hand only along a linear actuator to control the direction of the reaching motion. Even though the location of the hand is constrained to the linear trajectory, the subjects still can adopt own motor strategies (i.e., different joint kinematics) to compensate for the difference in their limb sizes or weakness of specific muscles. The subjects’ hands are fastened to a handle connected to a sliding block of the linear actuator during the experiment. Despite restricting upper limb motions to the linear motion, the position and orientation of the linear actuator should be freely adjustable in a three-dimensional space to implement reaching motions toward varying directions and a variety of initial postures. In the case of isometric force generation tasks, the position of the linear actuator should be still adjustable to locate the handle freely in accordance with various upper limb postures. Among in total five degrees of freedom (DOFs) required in the system, three of them determine the position of the linear actuator, and the other two adjust its orientation.

A three-DOF gimbal structure was adopted to the handle to adjust its orientation depending on the task and the subject since the hand orientation would affect the activation of upper limb muscles. Besides, subjects with neural injuries usually have twisted hands and flexed wrist due to abnormal contraction of the upper limb muscles. A six-DOF load cell is used to measure the force and moment applied to the handle by the subjects.

Target specifications of KULSIS were set in three aspects; the maximum allowable force, workspace of the handle, and mechanical stiffness. First, the maximum allowable force was set to 30 kgf. Except the extreme posture of raising an arm straight up, neurologically intact young men could generate the maximum force of 260 N to push or pull a handle (Das and Wang, 2004), which is smaller than the target maximum allowable force of KULSIS. Second, the workspace of the handle should be larger than 600 mm × 700 mm × 720 mm in anteroposterior, mediolateral, and superoinferior direction, respectively. These dimensions were chosen based on anthropometric data (Winter, 2009) by assuming a subject with a height of 1.8 m. The anteroposterior, mediolateral, and superoinferior lengths correspond to the gross length of upper arm and forearm (i.e., 33% of the height), 1.5 times of distance between the both shoulders (i.e., 26% of the height) and the vertical distance from pelvis to eye (i.e., 40% of the height), respectively. Last, mechanical stiffness should be equal to or larger than that of the existing comparable setups including KINARM (end-point, 16∼40 kN/m), HapticMaster (10∼50 kN/m) and MACARM (67∼80 kN/m).

### Design of Linear Actuator Positioner

**FIGURE 1:**
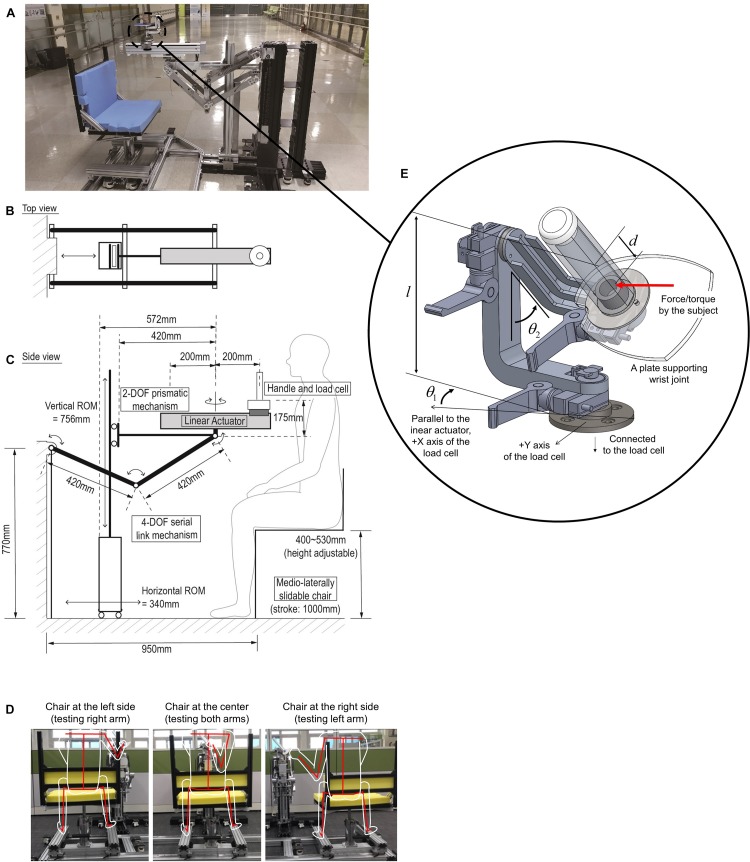
**(A)** Whole components of KULSIS. **(B,C)** Schematic of its structure in the top and the side view, respectively. **(D)** Single DOF sliding chair mechanism to determine medial-lateral position of the linear actuator to the subject (represented by white outlines, and a red skeleton). Depending on the relative position of the chair with respect to the linear actuator, either the right or left arm can be assessed. **(E)** The three-DOF gimbaled handle mechanism. The handle is mounted on the load cell. *l* and d represent the distance from the load cell to the rotational center of the gimbal and the distance from the rotational center to the point where the subject applies the force and the moment, respectively. θ_*1*_ and θ_*2*_ represent the angles of the first and the second gimbal joints, respectively.

The position of the linear actuator in the sagittal plane and its three-dimensional orientation was determined by a four-DOF serial link (RRRR) mechanism (Figures 1A–C). The two proximal axes determined the position of the actuator in the sagittal plane (i.e., anteroposterior and superoinferior positions). The lengths of the two proximal links of the RRRR mechanism were set to 420 mm. For fixing the position of the actuator firmly, a two-DOF prismatic (PP) mechanism was combined with the RRRR mechanism in parallel. The length of the horizontal link of the PP mechanism was equal to that of the second link of the RRRR mechanism so that the second link could rotate to the horizontal posture. Locking the prismatic joints fixed the position of the linear actuator. The other two axes of the RRRR mechanism rotated the linear actuator both horizontally and vertically to determine its orientation. The two joints were locked by clamping their axes. A hydraulic chair lift added one redundant DOF to adjust the superoinferior position of the linear actuator relative to the subject for the convenience of experimenters.

The mediolateral position of the linear actuator was determined by sliding the chair along a linear track (Figure 1D). From the center of the link mechanism, the chair could slide 400 mm in both directions. Also, the track was extended by an additional 200 mm in one direction for a safe seating of the subject. When the chair was positioned at the end of the longer side of the track, the subjects could sit on or leave the seat without interference of the linear actuator.

A ball-screw type linear motion module (RS-075N-Z05PR, Robostar, South Korea) was combined to a 100 W AC servo motor (APM-SA01ACN2, LS Mecapion, South Korea) to build the linear actuator. The motor includes an electromagnetic brake to fix the position of the handle. The maximum stroke of the module was 400 mm, which was a sufficient length to test the reach of the upper limb. The motor was controlled by a commercial motor driver (XSJ-230-06, Copley Controls, United States). The gross weight of the linear actuator including the load cell and the gimbaled handle was 10.3 kg. To reduce the gravitational load on the linear actuator positioner mechanism due to the weight of the links and the linear actuator, a gravity compensation mechanism based on passive springs was added to the RRRR mechanism. For the detail of gravity compensation, we recommend referring to Kim and Song’s study (Kim and Song, 2014).

### Design of Gimbaled Handle

The three-DOF gimbal structure was adopted to adjust the orientation of the handle bar where subjects’ hands were fastened during the measurements (Figure 1E). The gimbal mechanism was composed of two 90-degree-curved links and a bar. At the bottom of the bar, a plate was attached to support the wrist of the subject. The most proximal joint rotates the whole gimbal mechanism in the horizontal plane, and the next joint rotates the handle bar vertically. The most distal joint aligns the wrist supporting plate according to the subject’s wrist. The joints were locked by clamping the axes or unlocked to allow the rotation of the hand during the tasks. The gimbaled handle was designed to be light-weight (∼1 kg). Rib structures were adopted to increase the structural stiffness of the gimbal links. Magnetic encoders (SME360C-X05-W, Sera, South Korea) were used to measure the angles of the three gimbal joints (Table 1).

**TABLE 1:** Rotation (absolute value) of the linear actuator according to the directions of the loading (magnitude of 290 N) and the initial positions of the linear actuator.

**Direction of loading**	**Rotation at the initial position 1**			**Rotation at the initial position 2**			**Rotation at the initial position 3**		
			
	**X (deg)**	**Y (deg)**	**Z (deg)**	**X (deg)**	**Y (deg)**	**Z (deg)**	**X (deg)**	**Y (deg)**	**Z (deg)**
Post. (X)	0.01	0.66	0.02	0.01	1.16	0.03	0.04	1.28	0.01
Lat. (Y)	0.77	0.06	1.60	0.91	0.29	2.35	1.26	0.33	2.47
Inf. (Z)	0.10	1.57	0.17	0.02	1.78	0.06	0.09	1.62	0.04

*The three initial positions of the linear actuator correspond to the three postures of the linear actuator positioner shown in Figure 5.*

To measure the force and torque generated by the subjects, a six-DOF load cell (Delta SI-660-60, ATI Industrial Automation, United States) was attached between the gimbaled handle and the sliding block of the linear actuator. The force and torque applied to the handle bar (*F*_*x*_,*F*_*y*_,*F*_*z*_,*M*_*x*_,*M*_*y*_,*M*_*z*_) were reconstructed from the force and torque measured by the sensor (*f*_*x*_,*f*_*y*_,*f*_*z*_,*m*_*x*_,*m*_*y*_,*m*_*z*_) using Eq. 1. θ_1_ and θ_2_ represent the angles of the first and second gimbal joints, respectively. *l* and *d* denote the distance from the load cell to the rotational center of the gimbal and the distance from the rotational center to the point where the subject applies the force and the moment.

(1) $$\left[\begin{array}{c} F_{x}\\ F_{y}\\ F_{z}\\ M_{x}\\ M_{y}\\ M_{z} \end{array}\right]=\left[\begin{array}{cccccc} 1 & 0 & 0 & 0 & 0 & 0\\ 0 & 1 & 0 & 0 & 0 & 0\\ 0 & 0 & 1 & 0 & 0 & 0\\ 0 & \left(l-d\,c_{2}\right) & d\,c_{1}\,s_{2} & 1 & 0 & 0\\ -\left(l-d\,c_{2}\right) & 0 & d\,s_{1}\,s_{2} & 0 & 1 & 0\\ -d\,c_{1}\,s_{2} & -d\,s_{1}\,s_{2} & 0 & 0 & 0 & 1 \end{array}\right]\,\left[\begin{array}{c} f_{x}\\ f_{y}\\ f_{z}\\ m_{x}\\ m_{y}\\ m_{z} \end{array}\right]$$

where *c*_*i*_ = cosθ_*i*_, *s*_*i*_ = sinθ_*i*_ (*i* = 1, 2)

### Control Algorithms to Implement Varying Types of Reaching

**FIGURE 2:**
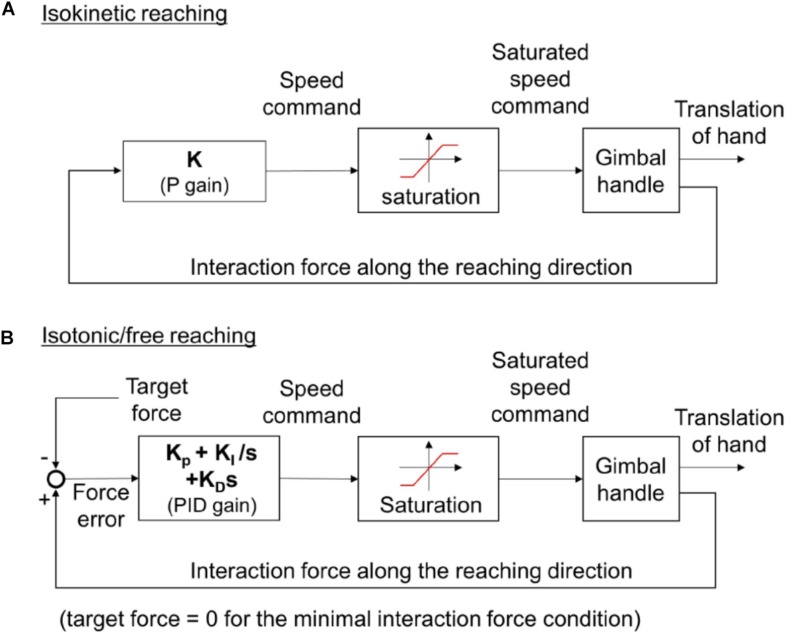
**(A)** The control algorithm for isokinetic reaching. **(B)** The control algorithm for isotonic and free reaching. The target force is set to be zero in case of free reaching. For the isokinetic reaching, the speed command is proportional (i.e., multiplied by a constant gain, *K*) to the magnitude of the interaction force that a subject applies to the handle. For the isotonic or free reaching, the speed command is determined by the PID control law from the force error, the difference between the force that a subject applies to the handle and target force. *K*_*P*_, *K*_*I*_, and *K*_*D*_ represent the proportional, integral, and differential gains, respectively.

KULSIS implemented three types of reaching motions; reaching in a constant speed (isokinetic reaching), reaching by generating a constant force (isotonic reaching), and reaching with a minimal interaction force between the subject and the KULSIS at the handle (free reaching). First, the isokinetic reaching is to generate the maximal force while reaching to a target distance at constant speed. The load cell beneath the handle measures the force applied to the handle. The actuator moves the handle at speed proportional to the magnitude of the force along the target direction (Figure 2A). The speed of the handle is saturated to the speed limit, set by the experimenter to evaluate the maximal force generation while the reaching at the constant speed. For the human experiment conducted in this study, if subject applied a force larger than 50 N along the targeted reaching direction, the speed approached to the limit of 20 mm/s (i.e., slope = 0.4 mm/s N).

Second, during the isotonic reaching, the subject exerts a constant force toward the target force direction regardless of the reaching direction (for example, keeping exerting a force anteriorly even the hand moves posteriorly). The speed of the handle is determined from the difference between the force applied by the subject and the target force magnitude set by the experimenter (Figure 2B). For instance, if a participant applies a force greater than the magnitude of the target force, the handle is accelerated. On the other hand, if the force is smaller than the target magnitude, the handle is decelerated. PID control law was used to produce the speed reference of the handle from the force error. In the literatures, to minimize the unnecessary interaction between the device and the subject, additional measurements such as EMG (Chen et al., 2016), motion tracking (Bai et al., 2017) or deflection at the motor shaft of a series elastic actuator (Kim and Deshpande, 2017) were used to detect and compensate the subject’s intent. In this study, however, the additional measurement was not adopted to reduce complexity of the KULSIS. Instead, the control gains were tuned so that the force error was kept less than 5 N while the subject push and pull the handle freely within the speed range of up to 200 mm/s. For more transparent actuation of the handle, an additional sensor may be applied.

The last condition, free reaching, can be implemented based on the same controller by setting the target force magnitude as zero.

## Evaluation of Kulsis

### Workspace of the Handle

**FIGURE 3:**
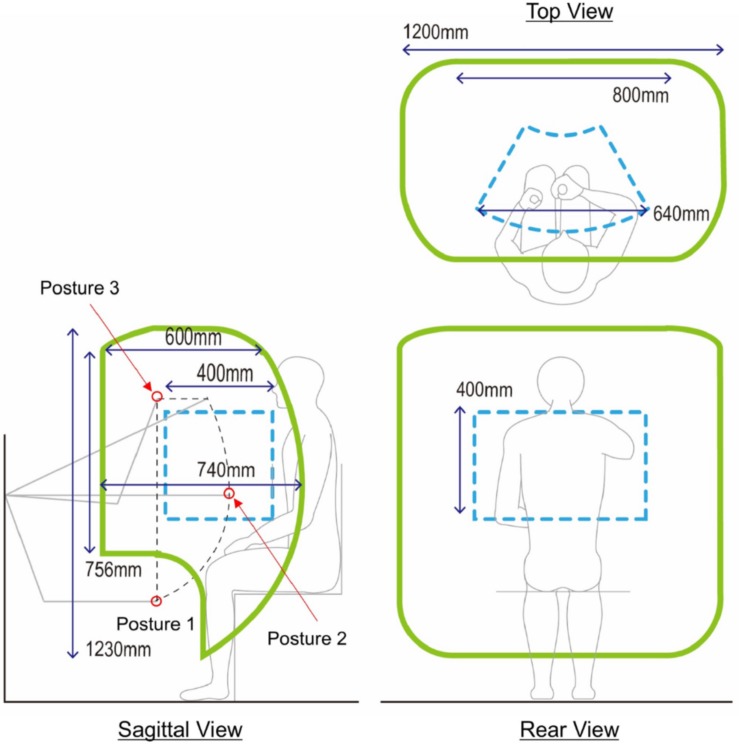
Workspace of the handle. The three green contours represent the workspace of the handle projected in each of three planes. In the sagittal view, the black dotted contour represents workspace of the linear actuator positioner. Gray lines represent the posture of the linear actuator positioner at each position. At the three positions marked as red circles, mechanical stiffness of the link structure was tested (see section “Mechanical Stiffness of the Linear Actuator Positioner”). Workspace of HapticMaster is represented as blue dotted contours for comparison in each of the three planes.

A large workspace of the handle guarantees that KULSIS as a testbed to examine intermuscular coordination at a diversity of more upper limb postures. The two factors that determine the size the workspace include the area in the sagittal plane and the length in the mediolateral direction. The sagittal work area (the green contours in the sagittal view of Figure 3) is expanded from that of the linear actuator (the black dotted contour), that is constrained by the RRRR mechanism and the PP mechanisms. First, regarding the vertical distance between the linear actuator and the handle, the work area translates 175 mm upward. Second, the work area expands by 200 mm both anteriorly and posteriorly since the handle can move 200 mm back and forth along the linear actuator. Also, the vertical rotation of the linear actuator at the third joint of the RRRR mechanism contributes to expanding the work area. For the mediolateral range of motion of the handle, by sliding the chair, the handle can be positioned up to 400 mm lateral from the center of a subject in both directions. If the linear actuator rotates horizontally by the last joint of the RRRR mechanism, the handle can move 200 mm further laterally. As a result, KULSIS has a workspace of which maximum dimensions are 740 mm (anteroposterior), 1200 mm (mediolateral), and 1230 mm (superoinferior) in a cartesian coordinate.

### Mechanical Stiffness of the Linear Actuator Positioner

#### Evaluation Methods

For reliable measurement, it is essential to maintain the position and orientation of the linear actuator. Deformation of the linear actuator positioner subject to an external force was measured by using a VICON motion capture system (VICON motion systems, Oxford, United Kingdom). According to the literature on accuracy of VICON system which used similar motion capture camera model and placement of the cameras compared to this study, mean position error of the stationary optical marker was 0.15 mm and variability of the position was lower than 0.025 mm (Merriaux et al., 2017). Based on this result, we concluded that VICON system is applicable to measure the deformation of the device of few millimeters. Specifically, a force of 290 N (i.e., the actual weight of ten 3 kgf weights) was applied as the maximum loading, comparable to the near maximal force generation capacity of healthy adults, to the sliding block of the linear actuator, located 150 mm posteriorly from the center of the actuator. The maximum value of loading was determined regarding maximal force generation capacity of healthy adults. The measurement was repeated for three directions of loading (i.e., posteriorly, laterally and inferiorly) and three different positions of the linear actuator (see posture 1–3 marked by red circles in Figure 3). The direction of the loading was controlled by hanging the weights through a cable routed by a pulley. Prior to the measurement, KULSIS was aligned to the reference coordinate frame of the motion capture system so that the positive *X*-, *Y*- and *Z*-axes of the reference coordinate were identical to the anterior, medial and superior directions of KULSIs, respectively. Three optical markers were located on the top surface of the linear actuator to define a three-dimensional coordinate frame for the linear actuator. Another two markers were attached to both ends of the third axes of the RRRR mechanism.

The mechanical stiffness was calculated as the magnitude of the loading (i.e., 290 N) divided by the deviation in the position of the linear actuator along the direction of the loading. The position of the linear actuator always most deviated in the direction of the loading. The deviation was quantified as position change of the center point of the two markers attached to the third axes of the RRRR mechanism. In addition to the mechanical stiffness, the deviation of the orientation was calculated as the rotation of the coordinate frame for the linear actuator in the form of *X*-*Y*-*Z* fixed angles around the reference coordinate frame.

#### Evaluation Results

The stiffness was in the range of 91.0∼1130 kN/m for the posterior direction, 32.6∼86.7 kN/m for the lateral direction and 102∼302 kN/m for the inferior direction depending on the initial position of the linear actuator. For the orientation of the linear actuator (see Table 2), the posterior and inferior loadings mostly produced the vertical rotation (*Y*-axis). The largest rotation produced by the posterior and inferior loadings was 1.78°. When the lateral loading was applied, the linear actuator rotates longitudinally (along *X*-axis) up to 1.26 deg and horizontally (along *Z*-axis) up to 2.47 deg.

**TABLE 2:** RMS error values of the calibrated force and moment at different gimbal postures and different directions of loading.

**Gimbal posture**	**Loading (up to 290 N)**	**RMS error**					
		
		**F_x_ (N)**	**F_y_ (N)**	**F_z_ (N)**	**M_x_ (Nm)**	**M_y_ (Nm)**	**M_z_ (Nm)**
1	Posterior(X)	6.95	1.81	34.57	0.17	1.21	0.13
	Lateral(Y)	1.31	4.09	3.96	0.68	0.10	0.16
	Inferior(Z)	5.49	2.44	6.53	0.32	0.61	0.20
2	Posterior(X)	6.29	3.78	28.18	0.39	1.21	0.17
	Lateral(Y)	4.71	4.68	2.83	0.66	0.48	0.12
	Inferior(Z)	4.29	2.35	6.99	0.27	0.33	0.15
3	Posterior(X)	12.08	1.38	12.49	0.15	1.30	0.39
	Lateral(Y)	6.16	3.81	3.85	0.46	0.66	0.27
	Inferior(Z)	11.25	0.96	5.71	0.15	1.20	0.35
Calibration (Gimbal posture 1∼3, loading up to 232 N)		5.31	2.05	3.85	0.27	0.55	0.19

*The last row shows RMS errors of the calibration based on data collected with the loading up to 232 N.*

### Accuracy of Force/Moment Measurement Through the Gimbaled Handle

#### Evaluation Method

The accuracy of force/moment measurement through the gimbaled handle was evaluated. When a subject applies a force and/or a moment to the gimbaled handle, the force and moment are transmitted to the load cell through the handle. The force and moment applied at the handle are then reconstructed from the force and moment measured by the load cell using Eq. 1. While forces of known magnitudes and directions were applied to the handle bar at a given position, load cell signals were collected. The measured force and moment were compared to their actual values. Specifically, we applied a loading ranging from 29 N (i.e., one 3 kg weight) to 290 N (i.e., ten 3 kg weights) along the three directions (i.e., posteriorly, laterally and inferiorly) at the point of the handle bar corresponding to the rotational center of the gimbal mechanism. The weight(s) and the handle bar were connected by a cable which was routed by a pulley to control the direction of the loading. Three different postures of the gimbal were tested; (θ_1_, θ_2_) = (0 °, 0°), (θ_1_, θ_2_) = (45 °, 0°) and (θ_1_, θ_2_) = (45 °, 90°). To compensate the sources of measurement error that could be generated while mounting it on the device, the load cell was calibrated with respect to the actual loading and the actual moment based on the least square method. It was assumed that the loading was applied exactly along the posterior, lateral or inferior directions as well as that magnitude of the force applied to the handle was equal to the weight of the weights. Then, the actual moment was estimated by multiplying the magnitude of the loading and the distance between the load cell and the point of action of the loading. Note that, for the calibration, we excluded the data collected when more than eight weights (i.e., a loading over 232 N) were applied since we observed the distortion of the gimbal structure and rapid change of the load cell signals if the posterior loading exceeded 232 N. After the calibration, the root-mean-square (RMS) error between the calibrated force/moment and the actual loading/moment was quantified. In addition, how accurately the moment at the handle can be reconstructed using Eq. 1 was also evaluated. Since we applied only a pure force to the handle, the desired value of the reconstructed moment was zero. The accuracy of the reconstruction was quantified as the magnitude of the reconstructed moment.

#### Evaluation Results

**FIGURE 4:**
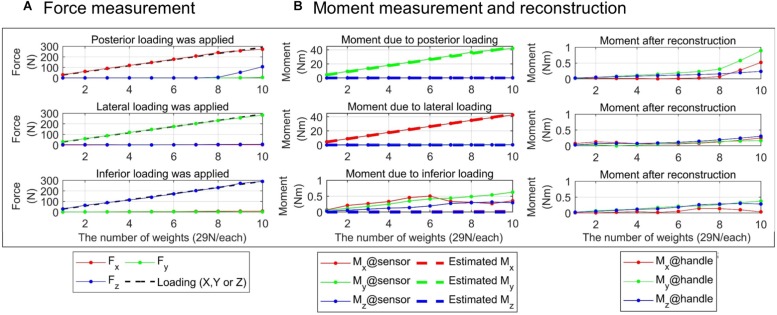
Evaluation of force/moment measurement accuracy. Result at the first posture of the gimbaled handle is presented. Absolute force/moment values in each of three directions are shown. **(A)** Forces measured by the load cell (left column). The black dotted line represents the actual loading applied on the cell. **(B)** Moment measured by the load cell (middle column, solid lines with star marks). The actual moment was estimated as the magnitude of the loading multiplied by the distance between the sensor and the point of the loading (dotted lines). Moment at the handle (right column) was reconstructed using Eq. 1.

Figure 4 shows the calibrated force/moment signals. The RMS error was 5.31 N, 2.05 N, 3.85 N, 0.27 Nm, 0.55 Nm, and 0.19 Nm for anteroposterior(*X*), mediolateral(*Y*) and superoinferior(*Z*) forces and moments around *X*, *Y*, and *Z* axes, respectively (Table 2). For the loading of 232 N in the three directions, the maximum force/moment error was 14.4 N, 4.64 N, 12.34 N, 0.65 Nm, 1.85 Nm, 0.64 Nm for the three forces and the three moments, respectively. When the posterior loading larger than eight weights (i.e., 232 N) was applied to the gimbaled handle, the superoinferior force increased rapidly (Figure 4A). As a result, a large RMS error of superoinferior force was observed at all the three gimbal postures. In addition to that, the RMS error of the anteroposterior force was also large when the posterior or inferior loading was applied at the third gimbal posture. The posterior and lateral loading applied at the handle bar produced a large moment about 43.5 Nm at the load cell. When the inferior loading was applied, a larger difference between the actual and measured moments was observed at the third gimbal posture compared to the other postures. The moment at the handle was estimated (see the right column of Figure 4). The reconstructed moment did not exceed 1 Nm in all cases. The RMS magnitude of the moment (0.24 Nm, 0.56 Nm, and 0.22 Nm for *X*, *Y*, and *Z* moments, respectively) was similar to that of the calibration error. Similar to the force, the moment also increased when the posterior loading exceeded 232 N at the first and second gimbal postures.

## Preliminary Intermuscular Coordination Assessment Using Kulsis

### Reaching Experiment With Healthy Subjects

**FIGURE 5:**
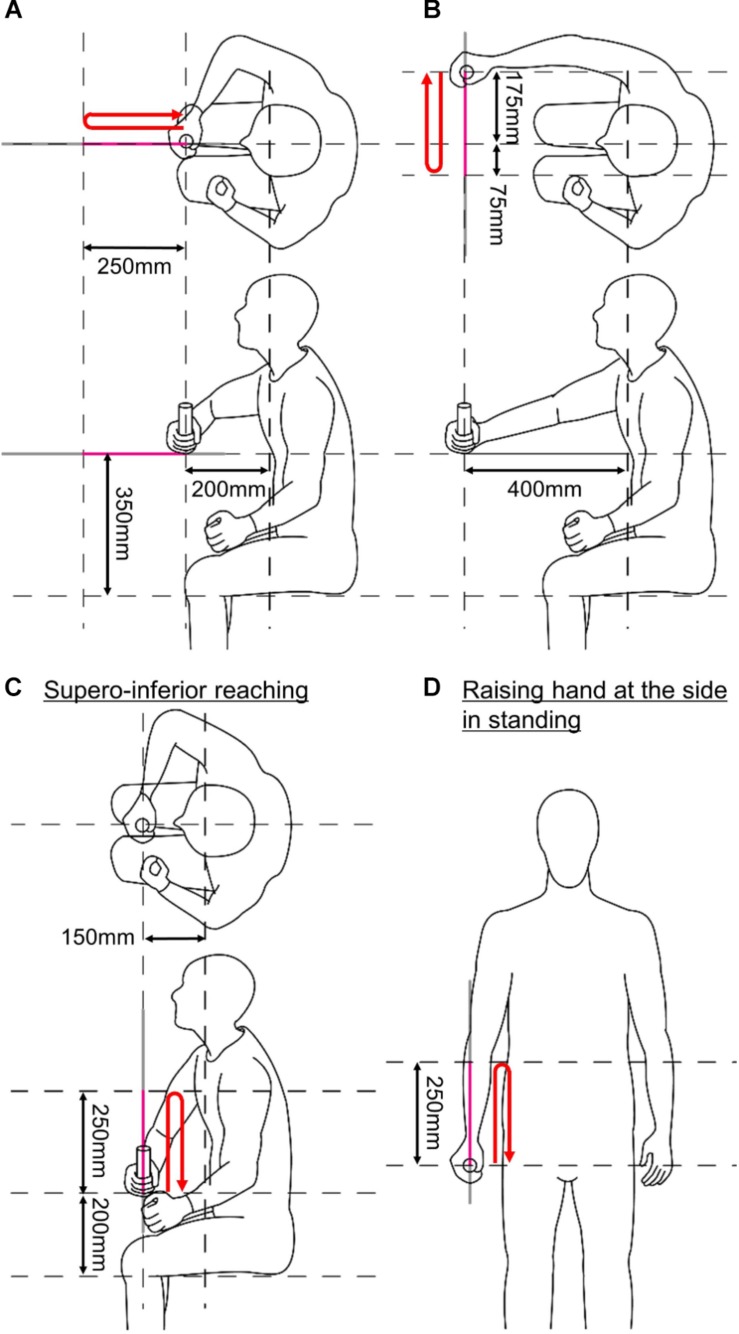
Initial postures of the four upper limb motions. **(A)** Anteroposterior reaching. **(B)** Mediolateral reaching. **(C)** Superoinferior reaching. **(D)** Raising a hand at the side in standing. Red arrows indicate reaching trajectories of a subject in each subplot.

Ten healthy subjects (five men and five women; age = 46.7 ± 3.97 years old; height = 169 ± 7.23 cm; and weight = 61.4 ± 10.5 kg) participated in this experiment, and their muscular activation was measured while performing isokinetic reaching tasks. EMG of twelve shoulder and elbow muscles (upper trapezius, lower trapezius, teres major, serratus anterior, clavicular fiber of pectoralis major, anterior deltoid, middle deltoid, posterior deltoid, triceps long head, triceps lateral head, biceps, and brachioradialis) was recorded using surface electrodes. For this experiment, we used a custom EMG measurement system (amplification gain, 1000; common mode rejection ratio, 120 dB; and bandwidth, 4000 Hz) based on the commercial differential amplifier (INA-128, Texas Instrument, United States). The subjects performed four gross motions of the upper limb (Figure 5). Since the custom EMG measurement system has analog outputs, the EMG signals were collected in synchrony with the force/position signals of KULSIS through a single data acquisition device (PCIe-6323, National Instruments, TX, United States).

The upper limb motions were selected from Wolf Motor Function Test (WMFT) (Wolf et al., 2001): anteroposterior reaching (reach and retrieve), mediolateral reaching (stacking checkers aligned along a line), superoinferior reaching (lifting a can), and raising a hand at side of the body in standing posture (lifting a basket). The tasks in the parenthesis correspond to the items of WMFT. The reaching started at the initial postures shown in Figure 5. KULSIS was aligned to the subject so that the subjects could hold the handle at those initial postures. For every trial, after reaching 250 mm, the subjects took a rest of 2 s at the final posture and then came back to the initial posture (see red arrows in Figure 5). They were asked to generate the maximum force along the reaching direction and to suppress the forces along the other directions. The experimental protocol was approved by Institutional Review Board of South Korea Advanced Institute of Science and Technology (KAIST IRB) and every subject gave written consent.

### Muscle Synergy Analysis

#### Analysis Procedure

Muscle synergies were analyzed from the EMG measurements. The surface EMG data were collected at 1000 Hz and processed in the following order; (1) low-pass filter with a cut-off frequency of 450 Hz, (2) band-rejection filter with cut-off frequencies of 55 Hz and 65 Hz to eliminate 60 Hz power noise, (3) high-pass filter with a cut-off frequency of 30 Hz, (4) rectification, and (5) low-pass filter with cut-off frequency of 0.3 Hz to obtain the amplitude of the EMG data. Based on the force and speed of the handle, the EMG intensity data were segmented into three phases; forward motion (i.e., from the initial posture to the final posture), rest at the final posture and reverse motion. The segmented EMG data were then resampled to have a uniform length across subjects and trials. Baseline EMG amplitude was subtracted. For each trial, the baseline amplitude was determined to be the smaller one between the average amplitude of the 2-s interval before the onset of the motion and the average amplitude of the 2-s interval of resting at the final posture. The processed EMG data were concatenated per subject. A NNMF algorithm (Lee and Seung, 2001) was applied to the concatenated EMG data to identify muscle synergies per subject. The appropriate number of muscle synergies was estimated as the minimum number of synergies that could account for over 90% of the total variance of the original EMG data (Roh et al., 2015). The muscle synergies obtained from all subjects were clustered based on a k-means clustering algorithm to group the synergies. The minimum number of clusters was used, that muscle synergies from one subject are not classified as the same cluster. All analyses were performed using MATLAB software (MATLAB R2018a, MathWorks Inc., United States).

#### Results

**FIGURE 6:**
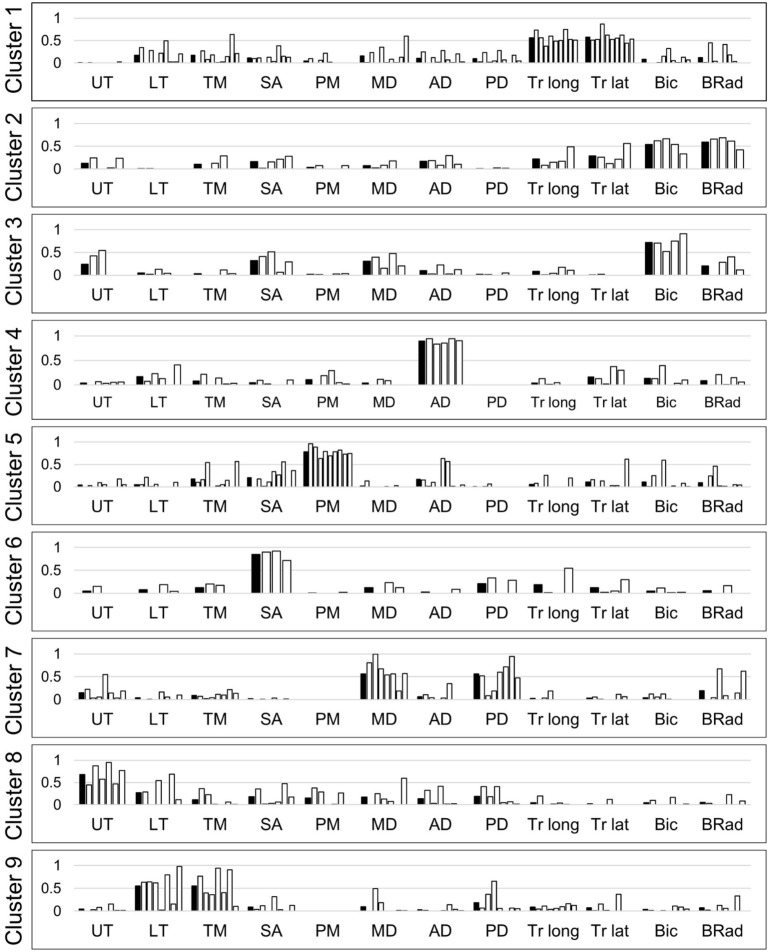
Nine clusters of upper limb muscle synergies. White bars represent muscle synergies identified from the data of individual subjects. Black bars represent the average of the muscle synergies (i.e., cluster centers). UT, upper trapezius; LT, lower trapezius; TM, teres major; SA, serratus anterior; PM, pectoralis major; MD, middle deltoid; AD, anterior deltoid; PD, posterior deltoid; Tr long, triceps long head; Tr lat, triceps lateral head; Bic, biceps; BRad, brachioradialis.

Per subject, four to seven muscle synergies were identified. A total of 55 synergies were obtained from ten subjects, and the synergies were classified into nine clusters (Figure 6). The first cluster was composed of elbow extensors (i.e., two heads of triceps) while the second and third clusters were mainly composed of elbow flexors (i.e., biceps and brachioradialis). The fourth cluster was dominated by the activation of the anterior fiber of deltoid which flexes the shoulder. The fifth cluster included the activation of the pectoralis major (the clavicular fiber), which flexes and adducts the shoulder. The seventh cluster was featured by the combination of the posterior and middle fibers of the deltoid, which abduct and extend the shoulder. The sixth, eighth and ninth clusters were combinations of back muscles moving the scapula. When the subjects reached their hands forward, cluster 1 (elbow extensor) and 4 (shoulder flexor) were employed. Co-activation of elbow flexors (cluster 2) was observed occasionally. On the other hand, activation of cluster 1 and 4 was reduced and cluster 2 was employed during the posterior reaching. Clusters 4, 5 (shoulder flexor and adductor) and 2 were activated for medial reaching while cluster 7 (shoulder abductor and extensor) was employed for lateral reaching. Cluster 5 was mainly activated for the superior reaching in sitting posture. When the subjects lower their hands, cluster 7 was activated. During raising a hand at the side of the body in a standing posture, clusters 7 and 3 (elbow flexor) were employed. Clusters 1 and 5 were activated when the hands went down to the initial height. Clusters 6, 8, and 9 (scapular muscles) were selectively activated during all motions to stabilize the shoulder joint.

## Discussion and Conclusion

### Performance of KULSIS

We developed KULSIS, a novel experimental setup for intermuscular coordination assessment of the upper extremity. It allows alignment of the force/moment-measuring handle to the hands in varying upper limb postures and straight reaching motions along a variety of three-dimensional directions. The specification of KULSIS was compared to that of the four comparable existing end-point type setups (Table 3). In the literatures, MACARM (Roh et al., 2012, 2013, 2015), HapticMaster (Ellis et al., 2009, 2016), and InMotion Arm (Tropea et al., 2013) were used for quantifying abnormal muscle coordination after neurologic injuries. In addition to them, other devices such as KINARM and WAM were included since they are potentially available for similar studies.

**TABLE 3:** Specification of various experimental setups for upper limb intermuscular coordination evaluation.

**Item**	**KINARM (End-point)**	**InMotion Arm**	**HapticMaster**	**WAM**	**MACARM**	**KULSIS**
Degrees of freedom	2(active)	2(active)	3(active)	6(active)	6(active)	1(active) + 5(passive)
workspace	400 mm, 760 mm (ellipse, 2D)	381 mm, 457 mm	400 mm (anteroposterior), 640 mm (mediolateral), 400 mm superoinferior)^∗^	A sphere with a diameter of 2000 mm	1600 mm (anteroposterior) 1400 mm (mediolateral), 2000 mm (superoinferior)	740 mm (anteroposterior), 1000 mm (mediolateral), 1230 mm (superoinferior)
Allowable maximum force	58 N (Peak)	45 N	100 N (Nominal), 250 N (Peak)	45 N	178 N^∗∗^	290 N (linear actuator) 232 N (gimbaled handle)
Mechanical stiffness	16∼40 kN/m	Not reported	10∼50 kN/m (depending on position of end-effector)	1500 kN/m	67∼80 kN/m	91∼1130 kN/m (anteroposterior) 32.6∼86.7 kN/m (mediolateral) 102∼302 kN/m (superoinferior) (Linear actuator)

*^∗^Horizontal work area of HapticMaster has a shape of an arc of inner radius of 280 mm, outer radius of 640 mm and angle of 1 rad(=57.3 deg). ^∗∗^It is the largest loading reported in literature at which MACARM was tested. At this force condition, it was reported that the workspace shrunk.*

There are three essential requirements in mechanical characteristics of the experimental setups for investigating intermuscular coordination patterns of human upper limb – the workspace, maximum allowable force, and mechanical stiffness. First, the workspace of KULSIS (i.e., 740 mm × 1200 mm × 1230 mm, in order of anteroposterior, mediolateral and superoinferior direction) covers most workspace of human upper limb in the sitting posture considering the length from the shoulder to the wrist, which is about 33% of the stature. The workspace of KULSIS is larger than that of HapticMaster (400 mm × 640 mm × 400 mm), which was reported as insufficient to cover workspace of the human upper limb (Sukal et al., 2007). However, it has smaller workspace than WAM (a sphere of 2000 mm in diameter) and MACARM (1400 mm × 1600 mm × 2000 mm).

Second, KULSIS has the largest maximum allowable force. KULSIS features a simple structure combining one active DOF (i.e., the linear actuator) to implement the straight reaching and five passive DOFs to set the position and the orientation of the linear actuator. The passive DOFs are fixed mechanically while the linear actuator implements various experimental tasks. The structural design allows KULSIS to maintain the position and the orientation of the linear actuator against the continuous external force up to 290 N, which can be generated by a healthy subject. Among the existing setups, MACARM can withstand the largest external force (i.e., 178 N) continuously. It was, however, reported that the end-point position of MACARM became unstable because of slackness of the cable mechanism at the larger portion of its workspace as the larger external force was exerted (Beer R. et al., 2008). However, having only one active DOF requires time to manually setup the position and orientation of the linear actuator and the gimbaled handle. When KULSIS was setup for anteroposterior, mediolateral, and superoinferior reaches, it took 219 (±98) s, on average.

Last, the mechanical stiffness of KULSIS was similar or larger than those of the most existing setups. For the loading smaller than 290 N, KULSIS would allow much smaller deflection than the other devices. Among the three directions of loading, KULSIS is the most vulnerable to the lateral loading. However, KULSIS can still be utilized to implement upper limb motor tasks in the lateral direction, since the human also has the smallest force generation capacity in that direction.

This study also verified that the accuracy of force/moment measurement by the gimbaled handle of KULSIS was not affected by varying postures of the gimbal mechanism and the extreme loading condition up to 232 N. If the loading exceeded 232 N, however, the force/moment measurement was affected presumably by the deformation of the gimbal structure. Among the existing devices, only MACARM reported its force measurement performance under an external loading condition (Beer R. et al., 2008). When a loading of 4.5 kgf (i.e., 44 N) was applied inferiorly to the gimbaled handle of MACARM, a force was measured up to 2% of the loading in the anteroposterior direction and up to 5% in the mediolateral direction. In case of KULSIS, when the loading of 232 N was applied along the three directions, the maximum error was 6.23, 2.00, and 5.32% for the anteroposterior, mediolateral and superoinferior forces, respectively. The moment at the handle was reconstructed successfully from the load cell measurements using Eq. 1. Even though the posterior and lateral loadings at the handle created the moments up to 43.5 Nm at the load cell, the reconstructed moment at the handle was smaller than 1 Nm even in the worst case.

In addition to the evaluation results above, KULSIS is also capable of implementing various types of upper limb motor tasks under various biomechanical constraints such as isometric force generation, isokinetic reaching, isotonic reaching, and free reaching. This feature will allow identification of comprehensive intermuscular coordination patterns rather than that specific to limited tasks and will contribute to investigation on generalizability of the intermuscular coordination of human upper limb.

### Evaluation of Intermuscular Coordination Using KULSIS

The averaged muscle synergies from clustering analysis were the elbow flexor, the elbow extensor, the shoulder flexor/adductor, the shoulder extensor/abductor and the combinations of the scapular muscles. The identified synergies verified that the neurologically intact subjects could control shoulder flexion/extension, shoulder abduction/adduction, and elbow flexion/extension separately. The composition of these patterns was similar to those reported in the previous literature which examined intermuscular coordination for isometric force generation tasks (Roh et al., 2012) and for various upper limb motions involving the shoulder and the elbow (Cheung et al., 2009). In particular, among the seven muscle synergies reported by Cheung et al. (2009), five synergies were also identified in this study except two synergies which are composed of muscles not measured in this study. However, differences in muscle synergy were also observed. Compared to Roh et al. (2012), the shoulder adductor/flexor synergy was divided into two separate synergies of the pectoralis major and the anterior deltoid in this study. However, according to Cheung et al. (2009), the pectoralis major was also identified as a separate muscle synergy. Compared to Cheung et al. (2009), the anterior deltoid was separated from the middle and posterior deltoids more clearly, but the biceps and brachioradialis were identified as one synergy in most subjects in this study. These patterns were close to those observed by Roh et al. (2012). We acknowledge that the comparison is based on a qualitative description mainly due to the differences in the task design and muscle selection between the current and previous studies.

### Limitations and Possible Design Improvements

Limiting the upper limb motions to a straight translation of the hand along the linear actuator can be considered as a limitation of KULSIS. To compare intermuscular coordination of different subject populations, such as participants with stroke versus neurologically unimpaired individuals, it is inevitable to restrict the trajectory of the hand to minimize the discrepancy of the end-point motion between the groups in comparison. For the current version of KULSIS, a linear actuator was used. Upper limb tasks in daily living can be tested with KULSIS by approximating the tasks to the closest straight motions as we tested four upper limb tasks. To implement more natural upper limb tasks without the approximation to the linear trajectory, a two-DOF planar mechanism may be adopted for the advanced version of KULSIS. If so, the trajectory of the hand can be controlled by designing a force field to suppress the movement of the handle off the desired path.

The position and orientation of the linear actuator were maintained against the external loading up to 290 N, but the force/moment measurement was affected by the distortion of the gimbaled handle when the loading exceeded 232 N. Mechanical stiffness of the gimbaled handle will be improved for more accurate force measurement under the extreme loading condition. To suppress bending of the gimbal links, a harder material such as steel can be added to the gimbal links to increase its structural stiffness. Gimbal joint structures will be re-designed to improve their sturdiness. These improvements will cause an increase of inertia of the handle. The heavier inertia can affect the control performance of the linear actuator, especially in a free reaching task. If the control performance is degraded despite optimizing control gains, we can apply an additional sensor to detect the subject’s intention.

## Conclusion

We propose KULSIS as a novel experimental setup for intermuscular coordination assessment in both isometric force generation and reaching tasks in the human upper extremity. Based on the quantitative, simultaneous measurement of motion and force as well as EMG, one can investigate how the alterations of the intermuscular coordination would affect their motor performance in the individuals with neural injuries. This paper mainly presents the design of KULSIS. Prior to the human study, its design was evaluated, and the parts that can be improved were identified. KULSIS features a large workspace which can cover that of the human upper limb and stable positioning of the linear actuator against the maximum loading of 290 N. The gimbaled handle structure will be modified to improve the accuracy of force/moment. Three different types of reaching tasks such as isokinetic, isotonic and free reaching as well as isometric force generation were developed for testing intermuscular coordination in diverse upper limb motor tasks. Overall, KULSIS can be used as an experimental setup for studying intermuscular coordination of human upper limb.

## Data Availability

The datasets generated for this study are available on request to the corresponding author.

## Ethics Statement

The studies involving human participants were reviewed and approved by the Institutional Review Board, Korea Advanced Institute of Science and Technology. The patients/participants provided their written informed consent to participate in this study.

## Author Contributions

J-HP, J-HS, JR, and H-SP conceived the concept of KULSIS. J-HP, HL, CP, and H-SP developed and evaluated the device, and carried out the experiment to identify muscle synergies of the healthy participants. J-HP and JR drafted the manuscript with inputs from all other authors. J-HS, JR, and H-SP contributed to the critical revision of the manuscript. HP supervised the study.

## Conflict of Interest Statement

J-HP, HL, CP, J-HS, and H-SP are inventors of the patent application (KR10-2019-0022360, pending), filed on February 26, 2019, for the proposed design of the force measurement device. The remaining author declares that the research was conducted in the absence of any commercial or financial relationships that could be construed as a potential conflict of interest.
